# Potential impact of efflux pump genes in mediating rifampicin resistance in clinical isolates of *Mycobacterium tuberculosis* from India

**DOI:** 10.1371/journal.pone.0223163

**Published:** 2019-09-26

**Authors:** Anshika Narang, Kushal Garima, Shraddha Porwal, Archana Bhandekar, Kamal Shrivastava, Astha Giri, Naresh Kumar Sharma, Mridula Bose, Mandira Varma-Basil

**Affiliations:** Department of Microbiology, Vallabhbhai Patel Chest Institute, University of Delhi, Delhi, India; Institute of Medical Sciences, Banaras Hindu University, INDIA

## Abstract

Despite the consideration of chromosomal mutations as the major cause of rifampicin (RIF) resistance in *M*. *tuberculosis*, the role of other mechanisms such as efflux pumps cannot be ruled out. We evaluated the role of four efflux pumps *viz*., MmpL2 (Rv0507), MmpL5 (Rv0676c), Rv0194 and Rv1250 in providing RIF resistance in *M*. *tuberculosis*. The real time expression of the efflux pumps was analyzed in 16 RIF resistant and 11 RIF susceptible clinical isolates of *M*. *tuberculosis* after exposure to RIF. Expression of efflux pumps in these isolates was also correlated with mutations in the *rpoB* gene and MICs of RIF in the presence and absence of efflux pump inhibitors. Under RIF stress, *Rv0194* was induced in 8/16 (50%) RIF resistant and 2/11 (18%) RIF susceptible isolates; *mmpL5* in 7/16 (44%) RIF resistant and 1/11 (9%) RIF susceptible isolates; *Rv1250* in 4/16 (25%) RIF resistant and 2/11 (18%) RIF susceptible isolates; and *mmpL2* was upregulated in 2/16 (12.5%) RIF resistant and 1/11 (9%) RIF susceptible isolates. This preliminary study did not find any association between Rv0194, MmpL2, MmpL5 and Rv1250 and RIF resistance. However, the overexpression of *Rv0194* and *mmpL5* in greater number of RIF resistant isolates as compared to RIF susceptible isolates and expression of *Rv0194* in wild type (WT) resistant isolates suggests a need for further investigations.

## Introduction

Rifampicin (RIF), one of the most important first-line anti-tuberculosis (TB) drugs, is a key factor in determining the effectiveness of treatment regimens [1, 2]. Since, more than 90% of RIF resistant strains are also resistant to isoniazid (INH), RIF resistance is used as a valuable surrogate marker for multidrug resistant (MDR) TB [3, 4]. The RIF resistant phenotype, in approximately 95% of the cases, is caused by spontaneous mutations, mostly located in the 81-bp core region (codons 507 to 533) of the *rpoB* gene, called the RIF resistance-determining region (RRDR) [5, 6]. However, in approximately 5% of clinical RIF resistant *M*. *tuberculosis* isolates, no mutations are found in the RRDR [7]. Absence of mutations in these isolates gives reasonable evidence on the role of other mechanisms in development of RIF resistance.

Other than mutations in the RRDR, resistance to RIF in *M*. *tuberculosis* could also be due to mutations in new, as yet unidentified loci, alteration in the target, change in drug permeability [8, 9], or involvement of efflux pumps which export various molecules outside the bacterial cell. The design of new therapeutic strategies may depend on the characterization of these efflux pumps. A few putative efflux pumps (*viz*. Rv1258c, Rv1410c, Rv1819c, PstB, Rv2936, Rv0783) have been observed to play a role in RIF resistance in *M*. *tuberculosis* [10, 11, 12, 13], however, the role of several other efflux pumps in RIF resistance still needs to be elaborated.

In the present study, we have explored the role of efflux pumps MmpL5, Rv1250, MmpL2 and Rv0194 in resistance to RIF by studying the expression of the genes encoding these membrane transporters under RIF stress, in *M*. *tuberculosis* isolates obtained from patients of pulmonary tuberculosis. The present study attempts to provide an insight into RIF resistance mechanisms in addition to chromosomal mutations.

## Methodology

### Ethics statement

Written informed consent and detailed history of contact were taken from each patient prior to the collection of samples, following approval of the study by the Institutional Ethics Committee of Vallabhbhai Patel Chest Institute.

### Bacterial strains and growth conditions

Clinical isolates (n = 130) of *M*. *tuberculosis* and the reference strain H37Rv were collected from the Department of Microbiology at Vallabhbhai Patel Chest Institute, University of Delhi, Delhi, India. The clinical isolates were obtained as part of a convenience sample obtained from new smear positive patients of pulmonary tuberculosis attending the Department of Respiratory Medicine at Vallabhbhai Patel Chest Institute which serves as a referral center for patients with respiratory diseases in North India. Cultures were grown on Lowenstein Jensen (LJ) medium slants and in Middlebrook 7H9 broth (Difco Laboratories, Detroit, MI) supplemented with OADC (oleic acid, albumin bovine, fraction V, dextrose, catalase) (Difco) and 0.2% glycerol at 37°C.

All the isolates (n = 130) were characterized by niacin, nitrate and catalase tests [14] and confirmed as *M*. *tuberculosis* complex (MTBC) by PCR Restriction Analysis (PRA) [15].

### Drug susceptibility profile

Indirect drug susceptibility testing to RIF was performed by the proportion method of drug susceptibility testing (PDST) for all the isolates, as described by the Revised National Tuberculosis Control Programme (RNTCP) of India [16]. The critical concentration of RIF used was 40μg/ml.

### DNA sequencing

The molecular drug resistance was confirmed by Sanger DNA sequencing performed by M/s I^st^ Base Asia, Malaysia for a subset of the isolates (41/130). DNA for sequencing was obtained from the same culture that was used for phenotypic RIF susceptibility testing.

### Selection of isolates for study of efflux pumps

On the basis of drug susceptibility test results and mutation studies, 27 clinical isolates (16 RIF resistant and 11 RIF sensitive) of *M*. *tuberculosis* were selected for further study.

The isolates were divided into two groups. Group I consisted of 5 RIF resistant and 5 RIF susceptible isolates that were screened for the activity of 10 efflux pumps in a preliminary study. Group II consisted of 11 RIF resistant and 6 RIF susceptible isolates tested for the activity of four efflux pumps (Table 1).

**Table 1:** Correlation between RIF MICs, *rpoB* mutations and efflux genes upregulated in Group I and Group II *M*. *tuberculosis* isolates under RIF stress.

	Isolate No.	PDST	MIC (mg/L)	Mutated codon and substitution	Efflux genes overexpressed
	**H37Rv**	S	0.0156	WT	*Rv1250*
Group I	**EP1-R1-13**	R	64	531 C>T	*mmpL5*
	**EP1-R2-13**	R	2	WT	*Rv1250*, *Rv0194*, *Rv1634*
	**EP1-R3-13**	R	4	WT	*mmpL2*, *Rv1250*, *Rv0194*, *Rv1273c*, *Rv1458c*, *Rv1877*, *Rv1634*
	**EP1-R4-13**	R	4	WT	*mmpL2*, *Rv1250*, *Rv0194*, *Rv1877*
	**EP1-R5-13**	R	2	531 C>T	*Rv1877*
	**EP1-S1-13**	S	0.0156	WT	*Rv1273c*
	**EP1-S2-13**	S	0.031	WT	*Rv1273c*
	**EP1-S3-13**	S	1	WT	*Rv1458c*, *Rv1877*
	**EP1-S4-13**	S	0.04	WT	*Rv1458c*, *Rv1877*, *Rv1634*
	**EP1-S5-13**	S	0.0156	WT	*Rv1273c*, *Rv1877*
Group II	**EP2-R1-15**	R	32	531 C>T	-
	**EP2-R2-15**	R	64	531 C>T	*mmpL5*, *Rv0194*
	**EP2-R3-15**	R	64	531 C>T	*Rv0194*
	**EP2-R4-15**	R	2	511 T>C	*mmpL5*, *Rv1250*, *Rv0194*
	**EP2-R5-15**	R	64	531 C>T	*mmpL5*
	**EP2-R6-15**	R	16	531 C>T	*mmpL5*
	**EP2-R7-15**	R	32	531 C>T	-
	**EP2-R8-15**	R	64	531 C>T	*mmpL5*
	**EP2-R9-15**	R	8	531 C>T	-
	**EP2-R10-15**	R	16	531 C>T	*mmpL5*, *Rv0194*
	**EP2-R11-15**	R	32	531 C>T	*Rv0194*
	**EP2-S1-15**	S	0.124	WT	-
	**EP2-S2-15**	S	0.00098	WT	-
	**EP2-S3-15**	S	0.0625	WT	-
	**EP2-S4-15**	S	0.125	WT	*mmpL5*, *mmpL2*, *Rv1250*, *Rv0194*
	**EP2-S5-15**	S	0.25	WT	-
	**EP2-S6-15**	S	2	WT	*Rv1250*, *Rv0194*

Table shows efflux genes which showed ≥2.5 fold increase in expression, relative to the non-exposed counterpart. Atleast 2.5 fold increase was considered as cut-off to denote significant overexpression. Increased expression in group I isolates was assessed using *sigA* as the internal control for RT-PCR. Group II isolates were assessed using two internal controls (*sigA* and *rrs*). Upregulation of an efflux gene in group II isolates was considered only if the enlisted efflux genes showed increased expression when normalized with both the internal control genes individually.

S-Susceptible; R-Resistant; WT-Wild type

### MIC determination

RIF, verapamil and Carbonyl Cyanide 3-Chlorophenylhydrazone (CCCP) were obtained from Sigma Aldrich (St. Louis, MO, USA). RIF and CCCP were dissolved in DMSO, and verapamil was dissolved in deionized water. 1000 mg/L stock solutions were freshly prepared for RIF as well as efflux pump inhibitors and filter sterilized (using 0.22μ filter) before use.

MIC to RIF was determined for the 27 *M*. *tuberculosis* isolates selected for study of efflux pumps. Verapamil and CCCP were tested for 12 clinical isolates (Table 2). MIC was determined by Microplate Alamar Blue Assay (MABA) and performed in 96-well U-bottom plates as described previously with minor modifications [17]. Growth controls (medium containing inoculum but no antibiotic) and sterility controls (medium without inoculum and antibiotic) were also included in the assay. Each concentration of drug and inhibitors was tested in triplicates and the procedure was repeated a minimum of three times. All the isolates for the present study were carefully selected and were the ones showing no variation in MICs of drug/inhibitor on repeated testing. The strains were considered as low level resistant (LLR) for RIF if their MIC ranged between 1–16 mg/L and high-level resistant (HLR) for RIF if the MIC was ≥ 32 mg/L [18].

**Table 2:** MICs of RIF in the presence and absence of efflux pump inhibitors.

	MIC (mg/L)			MIC (mg/L) of RIF in the presence of inhibitors	
Isolate No.	RIF	Verapamil	CCCP	Verapamil	CCCP
**H37Rv**	0.0156	120	0.1	0.0078	0.0156
**EP1-R1-13**	64	60	0.04	<4	<1.5
**EP2-R1-15**	32	60	0.07	8	32
**EP2-R2-15**	64	80	0.07	16	32
**EP2-R3-15**	64	40	0.09	<4	<1.5
**EP2-R4-15**	2	80	0.09	<0.25	2
**EP2-R5-15**	64	80	0.07	8	32
**EP2-S1-15**	0.124	80	0.11	<0.0156	0.0078
**EP2-S2-15**	0.00098	60	0.07	0.0078	0.0078
**EP2-S3-15**	0.0625	100	0.13	<0.0156	0.0156
**EP2-S4-15**	0.125	120	0.11	<0.0156	0.125
**EP2-S5-15**	0.25	60	0.1	<0.0625	0.0312
**EP2-S6-15**	2	60	0.07	<0.25	0.0039

MICs of verapamil and CCCP were individually calculated for all the isolates. Subinhibitory concentrations (1/2 MIC) of the inhibitors were used to supplement the medium. All the MICs were determined in triplicates in 3 or more biological replicates. The MIC values represented in the table are the ones consistent atleast 3 times.

MIC of RIF was also determined in the presence of efflux pump inhibitors verapamil and CCCP. MICs of RIF for all the isolates, in the presence of subinhibitory concentrations (1/2 MIC) of verapamil and CCCP, were determined as described previously [19]. The MICs were tested in triplicates atleast thrice to get a minimum of three consistent values.

### *In vitro* drug exposure to *M*. *tuberculosis* clinical strains and H37Rv

*M*. *tuberculosis* H37Rv and the clinical isolates were cultured in flasks containing Middlebrook 7H9 medium supplemented with 0.2% glycerol and 0.05% tween 80. The freshly growing culture of each isolate (10 ml) at mid log phase (OD600 ~0.5) was exposed to subinhibitory concentration of RIF (1/4 MIC) [20] (Table 1) and incubated at 37°C for 24 hours as described previously [21]. Unexposed culture was taken as control. Cell pellets were collected by centrifugation (8,000 rpm, 10 minutes, RT), washed once with 10 ml drug free medium, resuspended in 10 ml RNA protection buffer (Qualigens) and stored at -80°C as 1ml aliquots for RNA isolation.

### RNA Isolation and cDNA Preparation

RNA was isolated from exposed and unexposed cultures by using the RNeasy mini kit (Qiagen GmbH, Hilden, Germany), according to the manufacturer's instructions followed by DNase I (Thermo Fischer Scientific Inc., Waltham, MA) treatment as described previously [20]. The quality and quantity of the isolated RNA was determined by virtual gel electrophoresis on the DNR Bio-Imaging Systems (MiniLumi) and by spectrophotometric measurement (Tecan Infinite F200 Pro) of the A260/A280 ratio. The cDNA was synthesized using random hexamer primers provided with the First strand cDNA synthesis kit (Fermentas Life Sciences, Lithuania) according to the manufacturer’s instructions.

### Selection of efflux pump genes

Ten putative efflux genes (*Rv1273c*, *Rv1458c*, *Rv0194*, *Rv1819c*, *Rv1634*, *Rv1250*, *Rv1877*, *mmpL5*, *Rv3823c*, *mmpL2*) predicted as membrane transporters of *M*. *tuberculosis* H37Rv and selected on the basis of presence of high confidence mutations (HCM) in a multidrug resistant clinical isolate of *M*. *tuberculosis*, were included in the study [20]. The HCM were predicted after comparative analysis with the gene sequence of a MDR strain of *M*. *tuberculosis*, available at Open Source Drug Discovery [22].

### Generation of primers

Previously published primers were used for the efflux pump genes *Rv1273c*, *Rv1458c*, *Rv0194*, *Rv1819c*, *Rv1634*, *Rv1250*, *Rv1877*, *mmpL5*, *Rv3823c*, *mMmpL2* and for the internal control *sigA* [20]. Sequences of *rpoB* and the house keeping gene *rrs* were retrieved from http://genolist.pasteur.fr/Tuberculist/ [23] and the primers were designed using Gene Runner Version 3.01 software. The desired sequences were synthesized by Sigma Aldrich (INDIA). The primer sequences designed were as follows:

*rpoB* F 5’ AGACGTTGATCAACATCCG 3’

        R 5’ ACCTCCAGCCCGGCACGCTCACGT 3’

*rrs* F 5’ CGTCAGCTCGTGTCGTGAGATG 3’

        R 5’ GCATCGCAGCCCTTTGTACC 3’

### Real time expression analysis

Quantitative real time PCR (qRT-PCR) was performed to study the expression of putative drug efflux genes by using Quantitect SYBR Green Master mix kit (Roche Applied Science, Indianapolis, USA) in a Light Cycler 480 II real time PCR system (Roche Applied Science, Indianapolis, USA). The housekeeping sigma factor gene *sigA* and 16S rRNA gene *rrs* were used as internal controls in qRT-PCR assays [20, 24, 25]. Melt curve analysis was performed after each run in a LightCycler 480II instrument to confirm the specificity of the primers. Each qRT-PCR experiment was done on duplicate biological samples which were further assayed in triplicates. The starting amounts of cDNA for the amplification of putative efflux genes and the reference genes were equalized for each sample at 1.5 ng/μl. Relative quantification in clinical isolates was done to determine overexpression of efflux genes in cultures exposed to drug stress as compared to unexposed cultures, by 2-ΔΔCt method [26]. The data was analyzed using the built in quantification software. A relative expression equal to one indicated that the expression level was identical to the control, a fold change ≥2.5 was considered as significant overexpression.

### Data analysis

GraphPad Software (GraphPad Software Inc., La Jolla, CA, USA) was used to perform the Fisher’s Exact test. The difference in the number of susceptible and resistant isolates overexpressing efflux pumps was considered to be statistically significant if p <0.05.

## Results

### PDST and *rpoB* mutations

Of the 130 isolates of *M*. *tuberculosis* tested by PDST, 33 were found to be RIF resistant and 97 were RIF susceptible. Of these, presence or absence of mutations in the *rpoB* RRDR was confirmed by sequencing in 30 RIF resistant and 11 RIF susceptible isolates. Among the RIF resistant isolates, three did not contain any mutations in the *rpoB* RRDR while the remaining 27 isolates were found to have RRDR mutations.

The most common RRDR mutation was at codon 531 (22/27; 81.5%). This was followed by mutations at codon 526 (2/27; 7.4%) and one each at codons 511, 516, 518 (1/27; 3.7%). No mutations were observed in the RIF susceptible isolates.

### Spectrum of mutations in the *rpoB* gene of isolates selected for efflux pump study

To understand the significance of efflux pumps in RIF resistance, a subset of the resistant isolates having mutations at the RRDR of *rpoB* gene (n = 13), all the resistant isolates with no mutations at the RRDR of *rpoB* gene (n = 3) and all the sequenced susceptible isolates (n = 11) were included in the study group.

Among the 13 RIF resistant isolates with mutations, the mutation C>T at codon 531 was the most common, seen in 12/13 (92.3%) isolates. A single isolate, EP2-R4-15 had a mutation T>C at codon 511 (Table 1). No mutations in the RRDR of *rpoB* gene were observed in any of the RIF susceptible isolates.

### MICs of RIF and efflux pump inhibitors in the *M*. *tuberculosis* clinical isolates

MICs of RIF in the 16 RIF resistant isolates tested were ≥2 mg/L. Of these, 8/16 (50%) were low level resistant (LLR) with MIC ranging from 2–16 mg/L. The remaining (8/16; 50%) were high level resistant (HLR) with MIC ≥ 32 mg/L. MIC of 10 RIF susceptible isolates was ≤1 mg/L and one RIF susceptible isolate had MIC 2 mg/L (Table 1).

The MICs of efflux pump inhibitors were determined in a subset of isolates (6 RIF resistant and 6 RIF susceptible). The MICs of verapamil ranged from 40–120 mg/L and that of CCCP from 0.04–0.13 mg/L (Table 2).

### Correlation of mutations and MIC

On correlating the mutations at *rpoB* gene with MICs, we observed that most of the isolates with mutation C>T at codon 531 (8/12) had high level resistance to RIF (MIC ≥ 32 mg/L). The single isolate with mutation T>C at codon 511 had low level resistance to RIF (MIC = 2 mg/L). The resistant isolates with no mutations (n = 3) had MICs ranging from 2–4 mg/L (Table 1).

### Real time analysis of efflux pump genes

**Fig 1:**
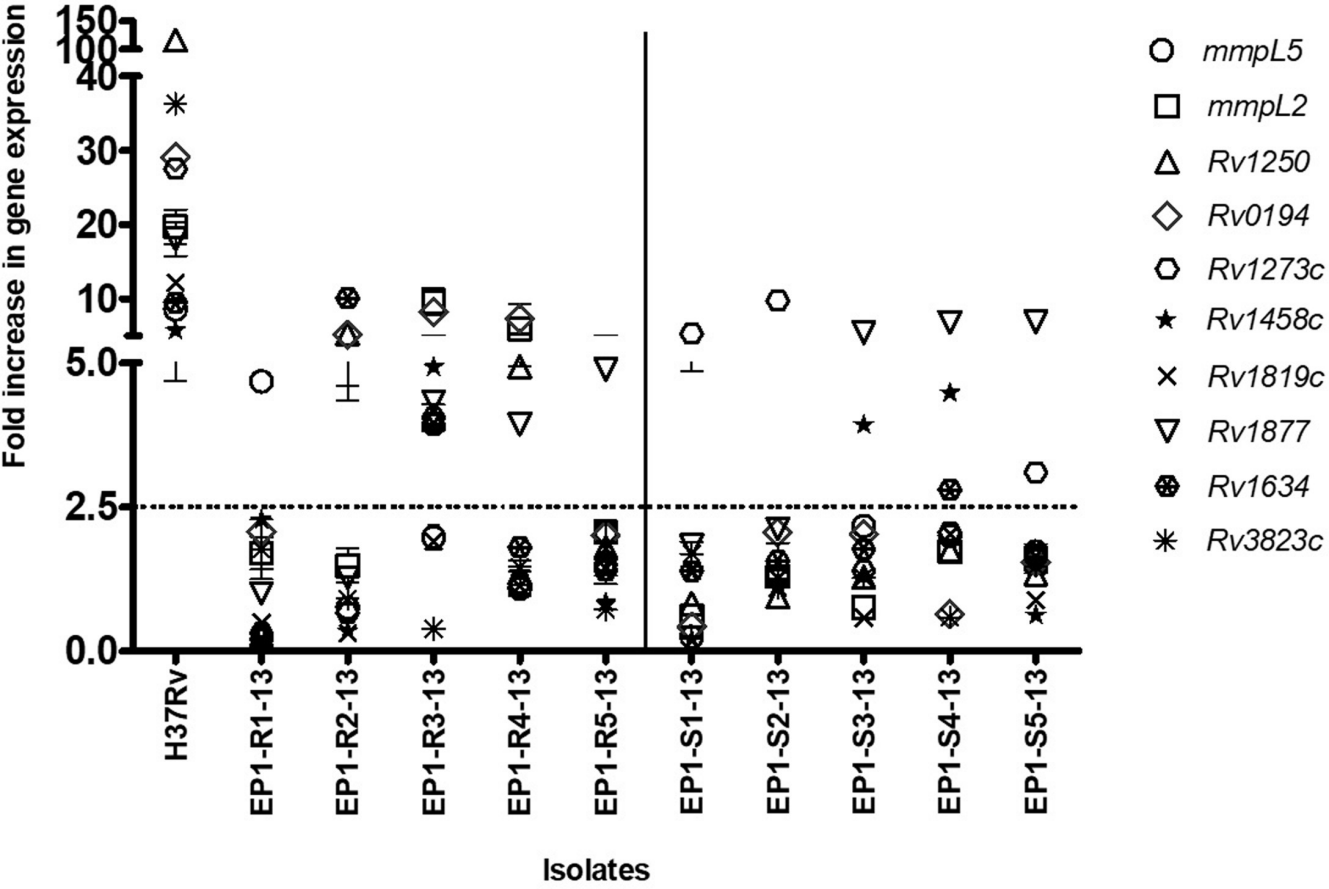
Relative expression of ten efflux genes in RIF resistant and RIF susceptible *M*. *tuberculosis* clinical isolates (Group I) exposed to RIF stress with their non-exposed counter parts, calculated by qRT-PCR.

**Fig 2:**
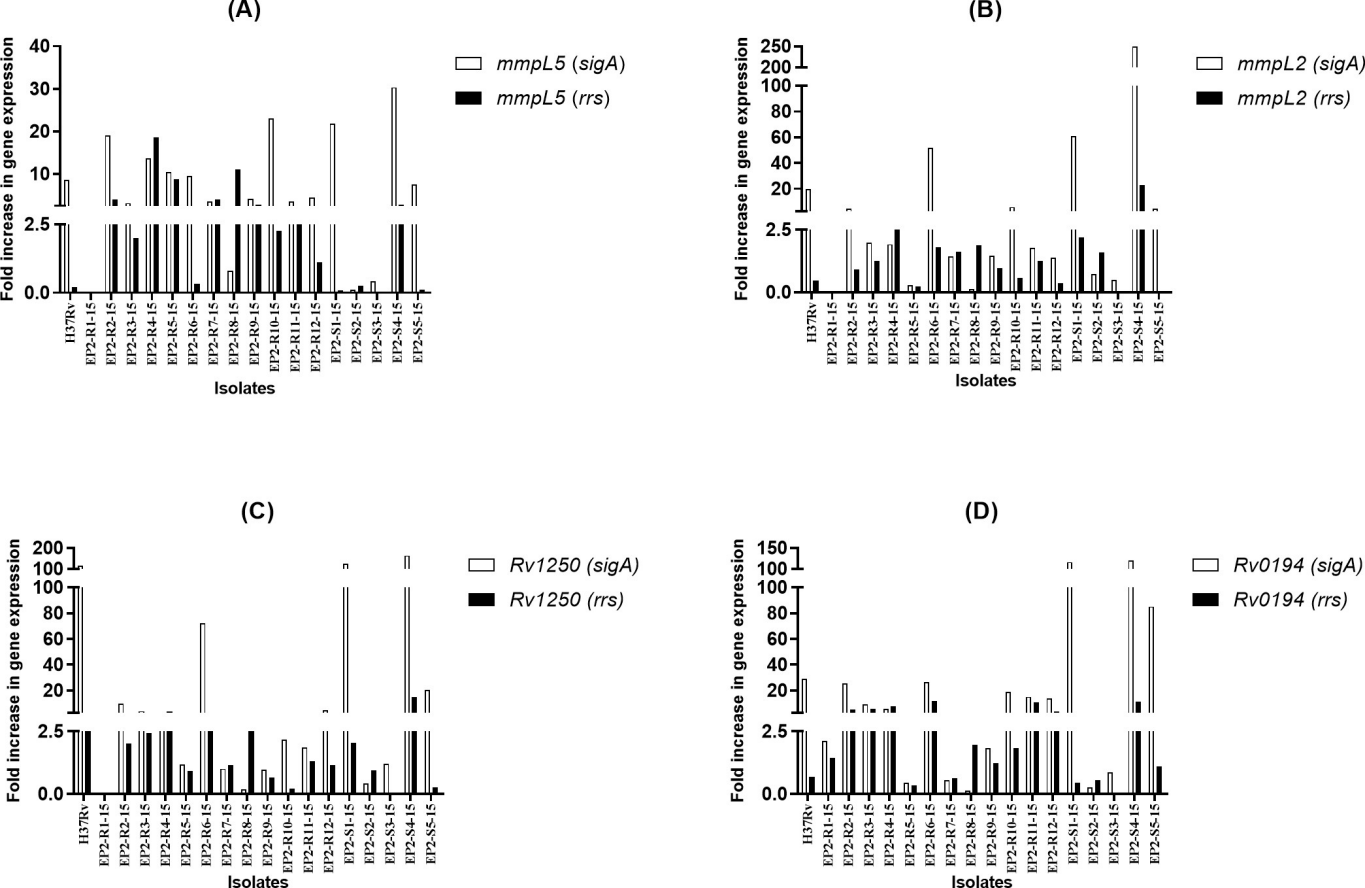
Relative expression of efflux genes in RIF resistant and RIF susceptible *M*. *tuberculosis* clinical isolates (Group II) exposed to RIF stress with their non-exposed counter parts, calculated by qRT-PCR: A. *mmpL5*, B. *mmpL2*, C. *Rv1250*, D. *Rv0194*.

In a preliminary investigation, the real time expression of efflux genes *Rv1273c*, *Rv1458c*, *Rv0194*, *Rv1819c*, *Rv1634*, *Rv1250*, *Rv1877*, *mmpL5*, *Rv3823c*, *mmpL2* was studied in Group I isolates consisting of five RIF resistant and five RIF susceptible *M*. *tuberculosis* isolates using *sigA* as the internal control. Of the five RIF resistant isolates, three showed increased expression of *Rv1250* and *Rv0194*. *mmpL2* and *mmpL5* were upregulated in two and one RIF resistant isolates respectively. None of the five RIF susceptible isolates showed significant increase in the expression of efflux genes *Rv0194*, *Rv1250*, *mmpL2* or *mmpL5* (Fig 1). The remaining genes were either expressed in more number of susceptible isolates (*Rv1273*, *Rv1458c* and *Rv1877*) or not expressed at all (*Rv1819c*, *Rv3823c*), except *Rv1634* which was overexpressed in greater number of resistant isolates though the difference was not statistically significant (p>0.05). Further, 11 RIF resistant and 6 RIF susceptible isolates were included in the study (Group II). The real time expression of the efflux genes showing upregulation in RIF resistant isolates in Group I *viz*. *mmpL5*, *mmpL2*, *Rv1250* and *Rv0194*, was studied in the additional isolates using *sigA* and *rrs* as the internal control genes. A gene was considered to be upregulated only if it showed increased expression when normalized with both the internal control genes individually. *mmpL5* was upregulated in 6/11 (54.5%) RIF resistant isolates as compared to 1/6 (16.6%) RIF susceptible isolates in Group II (p = 0.16) (Fig 2A); *Rv0194* was upregulated in 5/11 (45.4%) RIF resistant isolates as compared to 2/6 (33.3%) RIF susceptible isolates (p = 0.50). One of the susceptible isolates with significant upregulation of *Rv0194* had MIC 2 mg/L (Fig 2D). *Rv1250* showed increased expression in 1/11 (9%) RIF resistant and 2/6 (33%) RIF susceptible (MIC 0.125 mg/L and 2 mg/L) clinical isolates (p = 0.59) (Fig 2C). *Rv1250* was also upregulated in H37Rv. *mmpL2* was upregulated only in one susceptible clinical isolate and in none of the RIF resistant isolates (Fig 2B). Amongst the RIF susceptible isolates, one isolate, EP2-S4-15, showed upregulation of all the four genes *mmpL5*, *mmpL2*, *Rv1250* and *Rv0194* (Table 1).

The drug concentrations used for exposure were 1/4 MIC of RIF. *sigA* was used as an internal control. The experiment was performed in triplicates with two biological replicates. X-axis denotes the isolates used in the study. A line was drawn on X-axis between sensitive and resistant isolates. Y-axis denotes the fold increase in expression. Fold expression equal to 1 corresponds to no alterations in expression as compared with unexposed control. Fold change ≥2.5 in gene expression, relative to the non-exposed control denotes significant overexpression.

The drug concentrations used for exposure were 1/4 MIC of RIF. Two internal control genes used were *sigA* and *rrs*. The experiment was performed in triplicates with two biological replicates. X-axis denotes the isolates used in the study. Y-axis denotes the fold change in expression. Fold expression equal to 1 corresponds to no alterations in expression as compared with unexposed control. Fold change ≥2.5 in gene expression, relative to the non-exposed control denotes significant overexpression.

### Correlation between MIC, *rpoB* mutations and efflux pump gene upregulation

Amongst the Group I isolates, one was HLR to RIF and had a mutation (C>T) at codon 531 (EP1-R1-13). This strain also upregulated *mmpL5*. Group II had 7 isolates HLR to RIF. All these isolates had the mutation C>T at codon 531 of the *rpoB*. Of these, 5 showed upregulation of one or more efflux pump genes. Efflux pump genes *mmpL5* and *Rv0194* were each overexpressed in 3/5 HLR isolates.

Of the 4 isolates with LLR to RIF in Group I, only one isolate had a mutation C>T at codon 531, while all 4 showed upregulation of one or more efflux pumps. Amongst the Group II isolates with LLR (n = 4), one isolate had a mutation T>C at codon 511 (Table 1) and also overexpressed *mmpL5*, *Rv0194* and *Rv1250*. Mutation C>T at codon 531 was observed in 3 of the LLR isolates in Group II. *mmpL5* was upregulated in two of these isolates and *Rv0194* was upregulated in one isolate. The third isolate with a mutation C>T at codon 531 and LLR did not overexpress any efflux pump gene. One isolate (EP2-S6-15) was phenotypically susceptible to RIF. However, its MIC showed the isolate to be LLR to RIF. Though, the isolate did not have any mutations in the RRDR, it showed upregulation of *Rv0194* and *Rv1250*.

### Effect of efflux pump inhibitors on the MICs of RIF in the *M*. *tuberculosis* clinical isolates

To further examine the role of efflux pumps in RIF resistance, the changes in the level of resistance to RIF in the presence of efflux pump inhibitors verapamil and CCCP were studied in a subset of isolates (6 RIF susceptible and 6 RIF resistant) (Table 2). In all the RIF resistant isolates (n = 6), a minimum 4 fold decrease was observed in the MIC of RIF in the presence of verapamil. In the presence of CCCP, decrease in MIC was at least 2 fold in 4/6 RIF resistant isolates. Of the 6 susceptible isolates, 5 isolates showed >4 fold decrease in the MICs of RIF in the presence of verapamil. Addition of CCCP also led to at least 4 fold decrease in RIF MICs in 4/6 RIF susceptible isolates (Table 2).

## Discussion

The standard treatment for TB is a multidrug regimen that includes RIF and INH, two of the most efficacious drugs against *M*. *tuberculosis* infection. The development of resistance to these two drugs reduces the efficacy of standard anti-TB treatment by up to 77% [27]. The MDR phenotype is caused by sequential accumulation of mutations in different genes involved, due to inappropriate treatment or poor adherence to treatment [7, 28]. On the other hand, continued exposure of the organism to the drugs might lead to the activation of other mechanisms like efflux pumps which in turn help them resist the stress. In the present work, our focus was to study the efflux pumps related to RIF resistance in *M*. *tuberculosis*. The present investigation attempted to study efflux pumps as an additional mechanism leading to RIF resistance.

We first subjected a subset of isolates in the study to sequencing of the 81bp RRDR. Of the 16 RIF resistant isolates selected for the study (comprising of 5 RIF resistant isolates in group I and 11 in group II), 8 isolates showed high level resistance to RIF. All these isolates had mutation C>T at codon 531. The amino acid alterations at codon 531 have been seen to cause high level resistance to RIF by previous investigators also [2, 29].

It has also been observed, that mutations at codons 511, 516 and 522 cause low level resistance to RIF [18, 30]. The low level RIF resistant isolates in our study showed a varied spectrum of mutations. While one LLR isolate had a mutation at codon 511, three other isolates had no mutations. Surprisingly, unlike the observations of previous investigators [2, 29], 4 LLR isolates had mutations at codon 531.

To unravel the mechanisms of drug resistance in *M*. *tuberculosis*, many studies have elucidated the role of efflux pump genes using mRNA expression and DNA microarray analysis [31, 32, 33, 34]. In the present study, we analyzed the expression of efflux pump genes by qRT-PCR, in RIF resistant and RIF susceptible *M*. *tuberculosis* isolates after exposure to subinhibitory concentrations of RIF. Calgin et al have shown an increase in the expression of several efflux pump genes in the clinical strains which could be due to the exposure of anti-TB drugs during treatment of patients causing constitutive expression of efflux systems leading to increased MIC levels of the anti-TB drugs [35].

The efflux genes *Rv1273c*, *Rv1458c*, *Rv0194*, *Rv1819c*, *Rv1634*, *Rv1250*, *Rv1877*, *mmpL5*, *Rv3823c*, *mmpL2* were analysed in 5 RIF resistant and 5 RIF susceptible isolates, in a preliminary experiment (Group 1 isolates). Of the RIF resistant isolates, two isolates had a C>T mutation at codon 531 of *rpoB* gene. Surprisingly, three of the isolates, though resistant by PDST, did not have any mutations in the *rpoB* region. However, the MIC of these isolates was 2–4 mg/L. Several other investigators have also reported RIF resistant isolates without any mutations in the RRDR [36, 37, 38, 39, 40]. We observed that the efflux genes *mmpL5*, *mmpL2*, *Rv1250* and *Rv0194* were upregulated only in the RIF resistant clinical isolates in this initial study. This prompted us to increase the number of isolates studied. Hence, we added a set of 11 RIF resistant and 6 RIF susceptible isolates to the study and analyzed these for upregulation of *mmpL5*, *mmpL2*, *Rv1250* and *Rv0194* as well as *rpoB* mutations in the RRDR.

We also tested a subset of isolates (6 RIF resistant and 6 RIF susceptible) in Group II, for the effect of efflux pump inhibitors on the MICs of RIF. The efflux pump inhibitor verapamil led to reduction in RIF MICs of all the resistant and 5/6 of the susceptible isolates. Caleffi-Ferracioli et al have also shown that verapamil combined with rifampicin lead to downregulation of efflux pump related genes [41]. In addition, it has been shown previously, that verapamil can partially restore susceptibility to RIF in RIF resistant isolates [42]. It has also been demonstrated that the addition of verapamil to standard anti-TB chemotherapy increased the clearance of MDR *M*. *tuberculosis* strains in mice, thus highlighting the involvement of efflux pumps in drug resistance in *M*. *tuberculosis* [43]. CCCP also reduced RIF MICs in 4/6 RIF resistant and 4/6 RIF susceptible isolates. The reduction in MIC of RIF in susceptible isolates, evidenced a basal level of activity of efflux pumps in the *M*. *tuberculosis* isolates. Louw et al, [21] and Li et al, [34] also observed a reduction in MIC of RIF in RIF resistant isolates, with CCCP and reserpine.

Amongst the additional set of 11 RIF resistant *M*. *tuberculosis* isolates (Group II), all had a mutation in the *rpoB* gene. However, 8 of these also had one or more efflux pump genes overexpressed. It is possible that the remaining isolates with mutations had alternative efflux pumps active which were not analyzed in the present study. It is interesting to note that one isolate, EP2-S6-15, in group II was repeatedly found to be susceptible by PDST. It did not have any mutation in the *rpoB* gene, however, the region outside the RRDR was not sequenced in the present study. This isolate had MIC 2 mg/L and also showed upregulation of efflux genes *Rv0194* and *Rv1250*. The development of drug resistance is a sequential process. Efflux pumps maintain a low level of drug inside the bacterium, thus exposing the organism to a sublethal dose of the drug that induces the organism to develop resistance [44]. It is possible that the isolate EP2-S6-15, developed a low level resistance due to the presence of efflux pumps. It would be interesting to follow up such isolates to see if the propensity of the isolate to develop LLR, would also make it prone to develop mutations in the RRDR on exposure to anti-TB agents.

On studying the efflux pumps in these isolates and using 2 internal control genes to increase the stringency of our results, we observed that *mmpL2* was upregulated only in one susceptible isolate and in none of the RIF resistant isolates. Thus, its role in RIF resistance could not be established. *Rv1250* was upregulated in one RIF resistant and two RIF susceptible isolates. *Rv1250* had been shown to increase its expression in MDR-TB isolates under RIF stress in an earlier study [34]. However, in our study, in addition to one RIF resistant isolate two susceptible isolates also upregulated the gene. It was interesting to note, that Li et al [34] had used a single internal control in their qRT-PCR experiments, whereas we had increased the stringency of our assay by using two internal controls.

*mmpL5* was upregulated in 6 RIF resistant and one RIF susceptible isolate. We also observed that the RIF resistant isolates in which *mmpL5* was upregulated had a mutation in the *rpoB* gene, though the RIF resistant isolates showing overexpression of the gene were greater in number. *Rv0194* was upregulated in 5 RIF resistant and 2 RIF susceptible isolates. It is notable that, in our study *Rv0194* was also expressed in three WT RIF resistant isolates. In a previous study, overexpression of *Rv0194* had been shown to increase the MICs of ampicillin, vancomycin, novobiocin, and erythromycin for *M*. *smegmatis* [45]. The difference between the resistant and susceptible isolates in the expression of *Rv0194* and *mmpL5* in Group II was not statistically significant. Though, this is a preliminary study, limited to a small number of clinical isolates, overexpression of efflux pumps *Rv0194* and *mmpL5* in a greater number of RIF resistant isolates as compared to RIF susceptible isolates suggests a role in RIF resistance.

## Conclusion

Our study reiterated the importance of chromosomal mutations in RIF resistance. The study suggests that basal level RIF resistance imparted by the efflux pumps, more particularly, the role of MmpL5 and Rv0194 needs to be investigated further to gain additional insights into the mechanisms of efflux associated RIF resistance in clinical isolates of *M*. *tuberculosis*.
